# Midwives workload in the context of free maternal healthcare: a cross-sectional study based on the Workload Indicators of Staffing Needs (WISN) method in primary healthcare facilities in Kananga, Democratic Republic of the Congo

**DOI:** 10.1186/s12913-025-13656-y

**Published:** 2025-11-14

**Authors:** Paulin Nkolamoyo Musungula, Christophe Kalengo Nsomue, Emmanuel Esanga Longomo, Pascaline Muelu Mikobi, Bonard Djongesongo Djamba, Eric Mafuta Musalu

**Affiliations:** 1 Department of Health Organization Management, Higher Institute of Medical Techniques of Kananga, Kananga, Democratic Republic of Congo; 2 Institut de Recherche en Epidémiologie et Santé Publique, Kananga, Democratic Republic of Congo; 3 Faculty of Public Health, University of Kabinda, Kabinda, Democratic Republic of Congo; 4 Service of Service Pricing, Health Coverage Regulation and Control Authority, Kinshasa, Democratic Republic of Congo; 5 Provincial Health Division of TSHUAPA, Ministry of Public Health, Boende, Democratic Republic of Congo; 6 Department of Midwifery, Higher Institute of Medical Techniques of Kananga, Kananga, Democratic Republic of Congo; 7 Maternity of the Pax Polyclinic, Christian Medical Institute of Kasai, Kananga, Democratic Republic of Congo; 8 Central Office of the Ndekesha Health Zone, Ministry of Public Health, Kananga, Democratic Republic of Congo; 9 Department of Health Management and Policy, Kinshasa School of Public Health, University of Kinshasa, Kinshasa, Democratic Republic of Congo

**Keywords:** Workload, Midwives, Free maternal healthcare, Primary healthcare facilities

## Abstract

**Background:**

The Democratic Republic of the Congo has introduced free maternal halthcare to reduce direct payments made by households. This initiative may lead to an increase in the demand for maternal services, which could put significant pressure on healthcare providers. This study aimed to assess the workload of midwives and to estimate the pressure it exerts on these providers in this context.

**Methods:**

We conducted a cross-sectional study of 13 primary healthcare facilities in four health zones in the city of Kananga, including six first-level healthcare facilities and seven second-level healthcare facilities. The workload of midwives was assessed via the Workload Indicators of Staffing Need approach.

**Results:**

The basic services provided by midwives included childbirth, antenatal care, postnatal care and family planning. The results show that, in the seven first-level healthcare facilities, the number of midwives available was 20, whereas the actual need was 42, a shortfall of 22 agents. In the six second-tier healthcare facilities, 28 midwives were in post, whereas the calculated requirement was 35, a difference of 7 agents. Although some hospitals had a relatively balanced workforce, high average pressure (WISN ratio of 0.62) was reported for all hospitals studied. This pressure is moderately high in second-tier healthcare facilities (WISN ratio of 0.80) and very high in first-level healthcare facilities (WISN ratio of 0.48). All the primary healthcare facilities studied had only 44.8% of the midwives needed to provide free maternity services.

**Conclusions:**

The results of this study reveal a marked shortage of midwives in eight healthcare facilities. Five healthcare facilities currently have sufficient staffing levels, which highlights the relevance of extending the analysis to other facilities to assess the potential for a redistribution of human resources. Such an adjustment would help balance the workload and enhance the effectiveness of free maternal healthcare.

**Supplementary Information:**

The online version contains supplementary material available at 10.1186/s12913-025-13656-y.

## Background

The Democratic Republic of the Congo (DRC) remains an African country with a high burden of maternal and neonatal mortality, despite the progress made in recent years. According to the 2023–2024 Demographic and Health Survey (DHS-DRC) [1], the neonatal mortality rate recorded during the five years preceding the survey was 24 deaths per 1,000 live births. Maternal mortality, estimated at 746 deaths per 100,000 live births over the seven years preceding the same survey, remains far above the Sustainable Development Goal target of 70 deaths per 100,000 live births by the sustainable development goals. Among the identified causes of these maternal deaths are direct obstetric complications, notably postpartum hemorrhage, uterine rupture, hypertensive disorders, puerperal infections, and unsafe abortions [2]. These complications are often exacerbated by inadequate antenatal care and limited access to quality health services, which partly explains the persistence of maternal mortality [3].

Indeed, the proportion of births attended by skilled personnel rose from 81% in 2014 to 85% in 2024 [1]. However, fewer than one-third of women who gave birth received postnatal care within two days of delivery, including essential services such as fetal heart rate assessment and counseling on nutrition, breastfeeding, and vaginal bleeding. Coverage is particularly low (less than or equal to 8%) in the provinces of Bas-Uele, Kasaï Oriental and Lomami. During the same period, 16% of women did not receive any antenatal care (ANC) from the four recommended visits. The services delivered during these consultations typically include auscultation of fetal heartbeats, as well as counseling on nutrition and vaginal bleeding [1].

For postnatal follow-up of mothers, the DRC recommends three postnatal visits (PNCs) [1]. The first visit (PNC 1, 48 h after delivery) involves an assessment by midwives of both the mother and the newborn to identify potential complications and provide guidance on breastfeeding and neonatal care. The second visit (PNC 2, at 7 days post-partum) focuses on continued monitoring of the mother and the child and includes counseling on contraception, nutrition, and immunization. The third visit (PNC 3, before 6 weeks post-partum) aims to evaluate maternal recovery, monitor newborn development, and reinforce family planning as well as infection prevention measures. Unfortunately, 69% of women who gave birth did not receive any postnatal examination, contributing to the underuse of maternal healthcare [1]. This low utilization is often associated not only with the high cost of care [4] but also with other structural and sociocultural barriers, such as long distances to healthcare facilities, lack of transportation, unfavorable traditional beliefs and norms, limited decision-making autonomy of women, negative perceptions of care quality, and stigma related to certain maternal healthcare [5, 6]. These combined factors reinforce the underuse of maternal healthcare, even though adequate medical care is essential to reduce preventable maternal mortality [7].

To expand coverage of quality maternal healthcare—including childbirth, as well as antenatal and postnatal consultations—the DRC adopted a policy to subsidize maternal and neonatal health services in 2023 [8]. This policy, which has been piloted since 2021 in 11 provinces with World Bank support, aims to ensure equitable access to care [9].

In the DRC, this policy is implemented at the level of health zones (HZs), which are operational units responsible for delivering primary healthcare (PHC) and other secondary interventions to a defined population. At the primary level, each health zone includes two mandatory types of facilities: the health center (HC) at the first level and the general referral hospital (GRH) at the second level. The secondary and tertiary levels consist of provincial secondary hospitals and national hospitals and/or university clinics, respectively [10]. For accessibility reasons, optional healthcare facilities, such as health posts and reference health centers (RHCs), may also be established within an HZ. According to the Ministry of Public Health guidelines, HCs and health posts provide only a minimum package of activities, including the management of uncomplicated deliveries and other maternal services that do not require major obstetric surgery, for example [10].

Although some facilities currently perform interventions from the complementary package of activities typically managed by the general hospital or an RHC with a physician, HZs benefiting from free maternal healthcare in Kananga have been instructed to adhere strictly to their package of activities since the introduction of this policy. Consequently, this complementary package of activities is now exclusively available at GRHs. As a result, all obstetric complications are referred to these GRHs, concentrating the workload in these facilities. According to the same guidelines, maternal healthcare must be provided exclusively by qualified midwives.

In the DRC, these qualified midwives are state-certified graduates who have undergone three years of training at the Higher Institutes of Medical Technology, and A2 nurses retrained as midwives through a two-year training program. Their curriculum, aligned with the World Health Organization (WHO) and Ministry of Health guidelines, covers pregnancy, childbirth, the postpartum period, and the prevention of complications [11]. Other personnel, such as A2 nurses, sometimes assist with deliveries but have limited skills. There are also traditional birth attendants, without formal qualifications, who practice on the basis of personal experience, particularly in rural areas.

Therefore, midwives play a crucial role in reducing maternal and neonatal mortality, contributing to the success of free maternal healthcare [12]. In this context, their adequate availability and balanced distribution across healthcare facilities are essential to ensure safe and high-quality services [13]. Unfortunately, in 2022, the country had only 2,734 midwives for 14,827 healthcare facilities (HFs) and 7,472 health posts. In Kasaï Central Province, for example, there were only 197 midwives for 814 healthcare facilities, excluding the 762 health posts [14].

In several countries where similar policies have been implemented, generally positive results have been reported, including increased use of healthcare services [15–17] and improved working conditions for healthcare providers [18, 19]. However, in some regions of Ghana, Burkina Faso, and Tanzania, this increased demand for services has been accompanied by an increase in the workload of health personnel [20–22]. This is partly attributable to insufficient implementation planning and suboptimal allocation of human resources [23].

Since the abolition of fees for maternity services, a significant increase in the utilization of health services has been observed at the primary, secondary and tertiary levels of the Congolese health system. The rate of deliveries in HFs rose from 71% in 2007 to 83% between 2021 and 2022. In Kasaï Central, this percentage has reached 91% in 2022 [1]. Healthcare facilities in Kinshasa have also reported this increase [24]. Coupled with a shortage of qualified personnel, this growing demand for services may further increase the workload of health workers, thereby exposing them to occupational burnout and reducing job satisfaction, thereby compromising the quality and safety of the obstetric care provided [25, 26].

Although numerous African studies have documented the impact of free health care on the workload of health personnel, data remain limited or virtually nonexistent in the DRC. In a country where certain categories of personnel are underrepresented or absent in certain regions [14, 27], evidence to guide human resources for health (HRH) policies is imperative. The aim of this study was therefore to assess the workload of midwives and measure the pressure exerted on these agents in this context.

## Methods

### Context and study design

The study was conducted in the city of Kananga, the capital of Kasaï Central Province in the DRC. This city comprises five peri-urban health zones, corresponding to the five administrative communes, and covers a population estimated at over one million inhabitants. These five health zones include six GRHs, one secondary hospital, and approximately one hundred health centers.

**Fig. 1:**
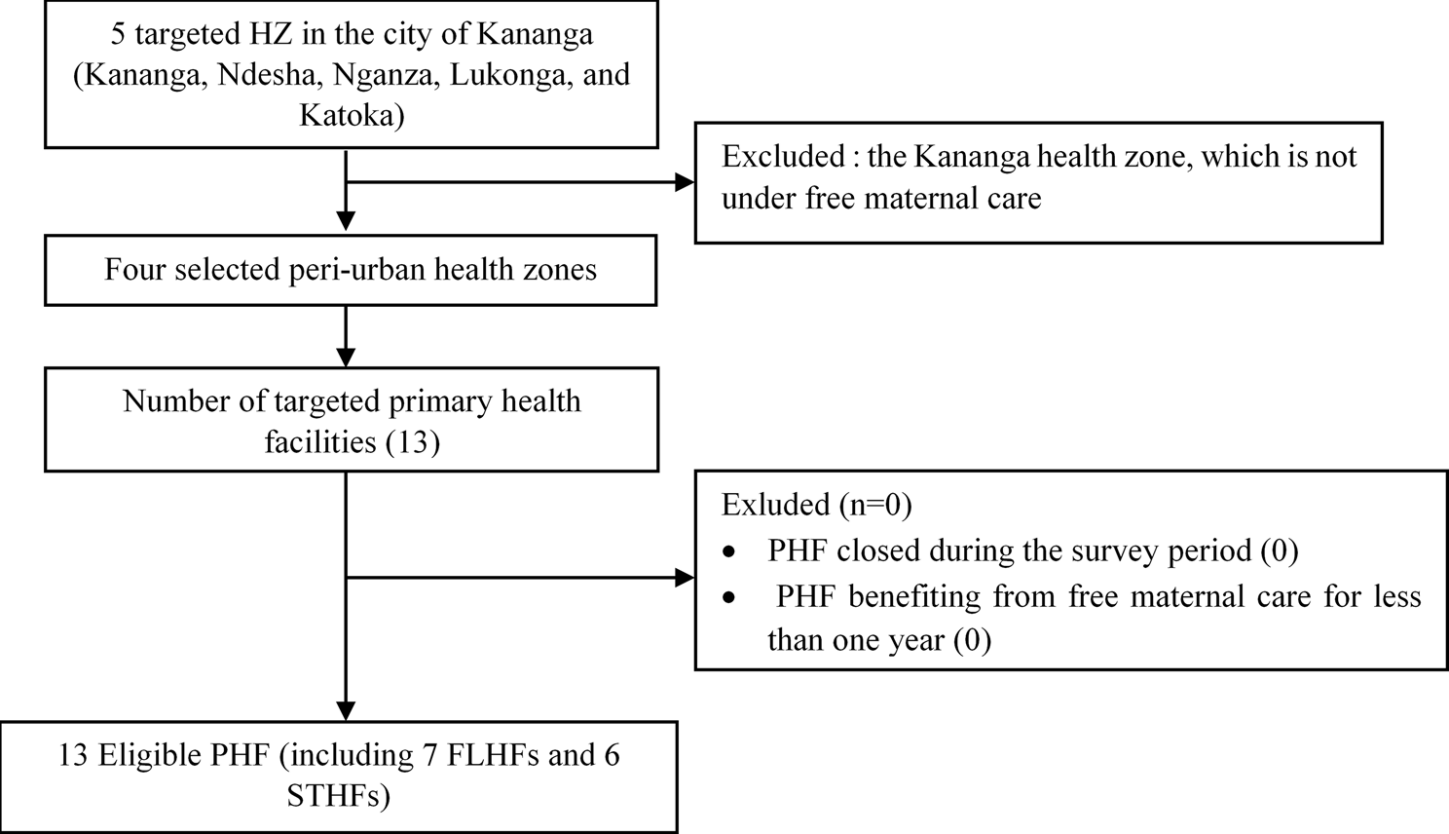
Sampling plan of the surveyed health facilities. HZ Health Zone, PHF Primary Health Facilities, FLHFs First-Level Health Facilities, STHFs Second-Tier Health Facilities

To obtain an overview of the workload of midwives, both in health centers (first level) and in general referral hospitals (second level), and considering the limited available resources, thirteen healthcare facilities were selected from four of the five health zones benefiting from free maternity services (see Fig. 1). In each HZ, two health centers (level I) and one GRH (level II) were included. The thirteen healthcare facilities studied included seven health centers, one secondary hospital and five GRHs; the two GRHs and one secondary hospital in the Tshikaji HZ were all retained. The thirteen primary healthcare facilities selected are as follows:

- Tshikaji HZ: Good Shepherd Hospital (GSH), Kalemba Mulumba SH (KMSH), General Referral Hospital Good Samaritan (GRHGS) and Saint Martyrs HC (MSHC);
- Katoka HZ: Saint-Georges GRH (SGGRH), Mamu Wetu HC (MWHC) and Sainte Famille HC (SFHC);
- Lukonga HZ: Lukonga General Referral Hospital (LGRH), Tudikolele HC (THC) and Saint Thomas HC (STHC);
- Ndesha HZ: Ndesha Etat General Reference Hospital (NEGRH), Christ the King HC (CKHC) and Shatshikumba HC (SHC).

More than two-thirds of these establishments are comanaged under public‒private partnerships between the Congolese state and the Protestant or Catholic churches.

We conducted a cross-sectional study via the workload indicators of staff need (WISN) quantitative approach; this method was developed and subsequently revised by the WHO to quantify staffing requirements [28]. It is a human resource management tool designed to determine the number of staff required to meet the workload of a health facility and to assess the level of work pressure experienced by its personnel. The method takes into account the annual volume of activities, the available working time per staff member, and the average time required to complete each task. In doing so, it provides a means of measuring both the supply of and demand for human resources within each health facility. Its implementation process comprises eight steps: (i) identification of the priority staff category and types of healthcare facility to be studied, (ii) estimation of available working time (AWT), (iii) definition of workload components, (iv) determination of activity norms, (v) establishment of standard workloads, (viii) calculation of allocation factors, (vii) determination of staffing requirements, and (viii) analysis and interpretation of WISN results.

### Study variables

Following the WISN method procedure, the workload of midwives was assessed via the following variables, whose definitions and measurement methods are presented in Table 1 [28].

**Table 1:** Study variables, their definitions, and measures

Variables type	Variables	Definition	Measure
Dependent	Workload/Staff Required	Number of midwives needed to perform all activities included in their current workload	Number of midwives required
Independent			
Workload components	Health Service Activities	Tasks performed by all midwives, for which annual statistics are routinely recorded (e.g., deliveries, antenatal and postnatal consultations, family planning, Prevention of Mother-to-Child Transmission of HIV [PMTCT])	Number of patients
	Support Activities	Tasks performed by all midwives, but for which annual statistics are not systematically collected (e.g., wound dressing, staff meetings, health education, report writing, shift handover)	Actual working time (per day/week/month/year)
	Additional Activities	Tasks performed by some midwives, usually without annual statistics (e.g., general administration, mentoring, supervision, review meetings)	Actual working time (per day/week/month/year)
	Activity Standard	Average time required to perform a service activity task, considering skills and local circumstances	Minutes per patient
	Available Working Time (AWT)	Time a midwife has available in a year to perform her tasks	Hours per year
	Annual Workload	Volume of service activities performed annually	Number of patients per year
	Annual Workload Standard	Amount of work a health worker can perform in a year if all available working time were devoted to a single activity	Number of patients per year
	Staff Available	Number of health workers currently in post	Number of midwives

HIV: human immunodeficiency virus; PMTCT: prevention of mother‒child transmission of HIV; AWT: available working time

### Data collection

Data collection was carried out over a six-month period: from April to June 2024 and then from January to March 2025. Using the WISN method, the data covered the year 2023 for Bon Berger Hospital and Kalemba Mulumba, the period from July 2023 to June 2024 for Ndesha État, and the year 2024 for the other facilities.

This data collection was based on a thorough review of staff administrative records and patients medical records. These paper documents were complemented by targeted interviews with midwives from the study facilities. The interview and observation guide, rigorously developed and specifically adapted to the research objectives, was designed using the standard data collection instruments recommended by the WHO in the WISN User Manual [28]. The English version of this guide, used to document the average duration of activities and to collect contextual information on AWT as well as annual activity statistics, is provided in Supplementary File 1 (Tables 1, 2, 3 and 4).

The collected data included the number of working days, daily schedules, and midwives absences extracted from attendance registers, the national calendar, individual personnel files, and official regulatory documents to estimate the AWT.

The activities corresponding to the three workload components were defined by a group of four experts in maternal health, public health, and human resource management, who received four and a half hours of training in the WISN process [28]. This group relied on reports, job descriptions, national standards, and WHO reproductive health guidelines. All identified activities were validated during plenary sessions, which enabled the development of the data collection tool. This tool was then pretested at Bon Berger Hospital, which was randomly selected from among the study facilities. The durations required to complete these activities were also defined by the experts, taking into account local circumstances. To ensure optimal precision, each activity was observed eight times in both first- and second-level facilities, allowing for the calculation of the average duration by level of care. Annual statistics the different service activities were obtained from maternity registers and triangulated with templates from the National Health Information System and monthly activity reports. Their reliability was strengthened by controls conducted by public utility institutions—Health Service Purchasing Funds. These institutions carry out quarterly verifications of services as part of the maternal health service purchasing mechanism.

### Data analysis

Using the WISN tool, we determined the number of midwives required on the basis of workload and estimated the pressure exerted on these staff.

The AWT was calculated in two steps. First, the theoretical number of working days per year was estimated by multiplying 52 weeks by 4 working days per week, in accordance with the working schedule of midwives in the HFs studied. The days of absence were then deducted from this theoretical total: public holidays (10), annual leave (30 days) [29, 30], sick leave, unauthorized absences, and training days (on average per health facility). The AWT in days was then converted into hours by multiplying by 8 working hours per day.

To estimate the required number of midwives, we first calculate the standard workload norm (SWN) for service activities by dividing the AWT by the average duration of each activity. The number of midwives required (A) for each activity, denoted from A1 to An, was then calculated by dividing the annual service statistics for each activity by its corresponding SWN. Next, we calculate the category allowance factor (CAF) for support activities (B) via the following formula: B = 1/[1 − (total percentage of time allocated to support activities/100)]. The daily percentage of time devoted to these activities was computed as (observed duration/8) × 100, where 8 represents the number of working hours per day. The individual allowance factor (IAF) for additional activities (C) was calculated by dividing the total individual allowance standard (IAS) by the TTD. Finally, the total number of midwives required (D) was estimated by the formula D = (A × B) + C. All calculations were performed via WISN software, version 2.2.170.1.

We used two indicators to interpret the WISN results obtained. The first is the difference between the current number and the required number of midwives. A negative difference indicates understaffing, a positive difference indicates overstaffing, and a zero difference reflects balanced staff. The second indicator is the WISN ratio, which is used to assess the pressure associated with the workload. A ratio below 1 indicates high pressure, a ratio equal to 1 indicates normal pressure, and a ratio above 1 indicates low pressure.

For descriptive purposes, the pressure exerted on midwives was classified into six levels: extremely high (ratio between 0.10 and 0.29), very high (ratio between 0.30 and 0.49), high (ratio between 0.50 and 0.69), moderately high (ratio between 0.70 and 0.89), normal (ratio between 0.90 and 1.19), and low (ratio greater than or equal to 1.20) [28]. In the case of shortage, we also expressed this pressure as a percentage via the following formula: pressure (%) = (1 – WISN ratio) × 100.

## Results

### AWT, workload components, and activity standards

The number of annual working days completed by midwives in the study facilities ranged from 131 to 162 days, corresponding to an available working time of 1,048 to 1,296 h (Table 2).

The health service activities of these staff included included deliveries, the first antenatal care visit (ANC 1), follow-up antenatal consultations (ANC 2 to ANC 4) and postnatal care, including three postnatal consultations (PNC 1 to PNC 3). Normal delivery, including labor monitoring, expulsion and immediate care of the mother and newborn, accounted for the largest proportion of midwives AWT. The time spent on these activities varied according to the level of the health facility. Despite the superior quality of their technical facilities, second-tier healthcare facilities (STHFs) spend more time on obstetric activities than first-tier facilities do. On average, the duration of a normal delivery was estimated at 278 h in Firts-Level healthcare facilities (FLHFs) and 345 min in STHFs. For assisted delivery, the average times observed were 289 and 397 min, respectively. Other service activities included family planning and prevention of mother‒child transmission of HIV (PMTCT). For family planning, the number of people who received advice was greater than the number who actually adopted a contraceptive method (Table 3).

**Table 2:** Available working time for midwives in the healthcare facilities examined

Health facility	Working days per week	Working hours per week	Annual leave	Public holidays	Sick leave	Special no notice leave	Training days per week	AWT in weeks	AWT in days	AWT in hours
GSH	4	8	30	10	3	0	5	40	160	1280
KMSH	4	8	30	10	3	2	5	39,5	158	1264
NEGRH	4	8	30	10	3	0	3	40.5	162	1296
LGRH	4	8	30	10	21	2	14	32,6	131	1048
SGGRH	4	8	30	10	5	2	4	39.3	157	1256
GSGRH	4	8	30	10	5	3	8	38	152	1216
THC	4	8	30	10	2	0	14	37.8	151	1208
CKHC	4	8	30	10	23	0	7	34.5	138	1104
MWHC	4	8	30	10	3	0	4	40.3	161	1288
SMHC	4	8	30	10	3	0	14	37.8	151	1208
STHC	4	8	30	10	8	0	14	36.5	146	1168
SFHC	4	8	30	10	3	0	12	38.3	153	1224
SEHC	4	8	30	10	5	3	12	37	148	1184

AWT Available Working Time, GSH Good Shepherd Hospital, KM SH Kalemba Mulumba Secondary Hospital, LGRH Lunkonga General Reference Hospital, SGHGR Saint Georges General Reference Hospital, GSGRH Good Samaritan General Reference Hospital, THC Tudikolele Health Center, Christ the King Health Center, MWHC Mamu Wetu Health Center, SMHC Saint Martyrs Health Center, STHC Saint Thomas Health Center, SFHC Sainte Family Health Center, SEHC Shatshikumba Etat Health Center

**Table 3:** Health service activities and health standards for midwives

Workload components	Service standards in minutes per patient	
	FLHF	STHF
Delivery (normal delivery)	278	345
Delivery (assisted)	289	397
Curative consultation		13
Antenatal clinic (ANC)—first visit	31	41
Antenatal clinic (ANC)—subsequent visits/revisits	22	31
Postnatal care (booked case and unbooked)	26	29
Family planning—counseling	18	21
Family planning—oral	7	9
Family planning—injectable	9	13
Family Planning - Introduction to the Use of Condoms	8	13
Family Planning - Instructions on how to use the menstrual cycle necklace method	11	18
Family planning—insertion (IUD and implant)	22	19
PMTCT—mothers (counseling for booked case)		21

FLHF Level healthcare facilities, STHF Second-Tier healthcare facilities, ANC antenatal consultation, PNC postnatal consultation, PMTCT prevention of mother-to-child transmission of HIV, IUD intrauterine device

The support activities identified included postoperative care (limited to second-tier healthcare facilities), staff meetings and auditing of benefits by the Public Utility Establishment Health Services Purchasing Fund (PUE-HSPF). Postoperative care consisted mainly of dressing changes twice a day. These activities were generally carried out on a weekly, monthly or quarterly basis (Table 4).

**Table 4:** Support activities and category allowance standards for frontline health workers for midwives

Workload components	Actual working time	
	FLHF	STHF
Dressing changes		2 h/month
Staff meetings	1 h/month	45 min/week
Group health education	31 min/week	30 min/week
Recording of Daily Data	1 h/day	1.5 h/Week
Handing over/taking over, report writing and ward round	41 min/week	52 min/day
Service Verification by PUE-HSPF	4 days/year	4 days/year

PUE-HSPF: Public utility establishment-health services purchasing fund

Additional activities were carried out mainly by maternity ward managers. These managers devoted a significant proportion of their time to the general administration and supervision of trainees. Tasks such as supervising junior staff and reviewing maternal deaths took up relatively less time (Table 5).

**Table 5:** Additional activities and individual allowance standards for health workers

Workload components	Actual working time		
	Number of staff performing the task	FLHF	STHF
General administrator	1	2 h/week	4 h/week
Monthly Report Writing	1	1.5 h/month	5 h/month
Mentoring of Subordinates	1	30 min/week	1 h/week
Monitoring Meeting	1	4.3/Month	6 h/month
Meeting for the Review of Maternal Deaths	1		3 h/month
Meeting of Department Heads	1		2 h/week
Supervision of Students	1	2 h/week	3 h/week

### Calculation of staffing requirements and workload pressure for midwives

Using the validated variables in Tables 3, 4 and 5, we applied the WISN method to calculate the number of midwives required for the healthcare facilities studied. As shown in Table 6, a total of 48 staff assigned to the studied maternity units were available, all with the same working hours and TTD. Among them, 37 (77%) were qualified midwives holding a Graduat diploma (the former higher education system in the DRC), whereas the remainder were hospital nurses trained in emergency neonatal care (ENC). The calculated actual need was estimated for 77 midwives, resulting in a shortfall of 29 staff members. These staff are under high average workload pressure (average WISN ratio of 0.62). Although three healthcare facilities (GSH, SGGRH and SMHC) were in balance and two others (NEGRH and GSGRH) had a surplus, midwives in eight understaffed healthcare facilities faced high workload pressure. This pressure was moderately high in the STHFs (WISN ratio of 0.80) and very high in the FLHFs (WISN ratio of 0.48).

As the final total number of staff required included decimal values, we applied the rounding rules for fractional numbers as outlined in the WISN user manual [28]. Table 5 in Supplementary File 2 shows the rounding rules based on the WISN manual.

**Table 6:** WISN results for midwives in health care facilities

Health facility	Current Number (A)	Required Number (B)	Shortage or excess	Staff issue	WISN Ratio (A/B)	Workload pressure (%)	Workload pressure
GSH	4	4	0	Balance	1		Normal
KMSH	4	7	-3	Shortage	0.57	57.1	High
NEGRH	6	4	2	Excess	1.50		Low
LGRH	4	11	-7	Shortage	0.36	36.4	Véry high
SGGRH	6	6	0	Balance	1.00		Normal
GSGRH	4	3	1	Excess	1.33		Low
THC	2	7	-5	Shortage	0.29	28.6	Extremely high
CKHC	3	8	-5	Shortage	0.38	37.5	Very high
MWHC	4	8	-4	Shortage	0.50	50.0	High
SMHC	2	2	0	Balance	1.00		Normal
STHC	4	6	-2	Shortage	0.67	66.7	High
SFHC	3	6	-3	Shortage	0.50	50.0	High
SEHC	2	5	-3	Shortage	0.40	40.0	Very high

## Discussion

The DRC has introduced a free maternity policy to reduce the burden of direct payment for services on households and increase coverage of maternity services. This initiative is likely to increase demand for services, which could put enormous pressure on providers. It could also promote the occurrence of informal payments. For example, a study conducted in Kinshasa reported that [24], although more than 90% of women reported receiving fully free care in supported healthcare facilities, 9% (95% CI: 7.8–10.6%) indicated that they had made a payment, of which 28% were for transfer cases and 16% for cesarean sections. These findings suggest that, even under free maternal healthcare policies, certain complex or exceptional procedures may still give rise to informal payments.

Our study focused on midwives, who are the main providers of maternal health care. Since the introduction of free maternal healthcare, donors, through the Ministry of Health, have required that maternal healthcare be provided exclusively by qualified midwives. In the healthcare facilities studied, this requirement was strictly adhered to only at the Good Shepherd Hospital. Among a total of 48 available staff, 37 (77%) were qualified midwives, whereas the remaining 13% were hospital nurses trained in ENC. Integrated into the midwife category, these nurses in twelve of the thirteen studied facilities are assigned to maternity units and perform the same tasks as certified midwives do. As previously mentioned, some studies [20, 31] assessing the workload of health personnel who combine midwives and nurses under a common standard of practice may indicate that there are not necessarily significant differences in the services they provide.

The results of this study highlight the limited number of midwives available to meet the current workload in the healthcare facilities studied, particularly in health centers, compared with hospitals. Although some facilities have a balanced staffing level, nearly two-thirds (61.5%) operate with only 44.8% of the required workforce. Overall, a shortage of 29 staff members was observed, with high average workload pressure (WISN ratio of 0.62). Evidence from the literature indicates that the removal of user fees for maternal healthcare generally leads to increased demand, thereby exacerbating the workload of already understaffed providers [22, 32, 33]. In our context, it is therefore plausible that a potential increase in demand for maternal healthcare could further exacerbate this imbalance, although no direct data currently confirm this.

While this policy remains a laudable measure to facilitate maternal care coverage in the DRC, it may not have been sufficiently accompanied by human resource reinforcement. This inadequacy of staffing levels is part of the realities of the DRC, where HRH is more concentrated in urban areas. In carrying out this study in predominantly urban HFs, we expected to find a sufficient number of midwives. Unfortunately, their low presence in the labor market, compounded by poor geographical distribution and an unresponsive recruitment system, further exacerbates this situation in the region [14]. This uneven distribution results in a high concentration of health workers in urban centers, where security, infrastructure, financial opportunities, and other incentives are better, whereas rural and conflict-affected areas experience severe shortages [34, 35]. For example, a study conducted in Ituri showed that only 7.2% of midwifery positions were filled in rural districts, whereas 24.9% were filled in peri-urban areas [36].

Quality maternity care requires not only physical access to services but also the presence of competent and motivated staff in a favorable working environment [33]. As pillars of maternal health, these overworked midwives are at risk of burnout, less job satisfaction and, ultimately, disengagement [25, 26]. Clearly, this can have an impact on the quality and quantity of services provided, leading to difficulties in managing obstetric complications, less time to complete tasks, compromised quality of care and difficulties in reducing maternal mortality. This may also hinder progress toward universal health coverage (UHC) in the DRC [37]. However, free maternal healthcare, as the initial component of UHC, was established to respond rapidly to health needs and save lives.

We emphasize the imminent need for midwives in the HFs of the city of Kananga. In this case, the use of WISN would make it possible to objectify the workload and determine the real staffing needs of each HF [28]. Our results are comparable to those reported in Tanzania, parts of Burkina Faso and Niger, where a severe shortage of perinatal providers (nurses and midwives) has been identified [20–22].

As noted above, the midwives in twelve of the healthcare facilities studied share their tasks with nurses trained in Emergency Neonatal Care, due to their wide range of responsibilities and very limited numbers. In this case, the Ministry of Public Health could consider the use of task-sharing with other professional categories to reduce the workload of midwives, provided that this is accompanied by adequate training and ongoing supervision mechanisms [38].

The results of the WISN method provide reliable evidence to inform decisions about HRH [39]. The implementation of the WISN process at the first and second levels of PHC in this region, together with evidence of a shortage of midwives in the HFs studied, provides an opportunity to integrate the WISN method into policies, strategies and processes for planning and managing HRH in the DRC.

If these urban facilities, which are supposed to have sufficient HRH, are already understaffed in terms of midwives, it is reasonable to assume that work overload is even more marked in HFs in disadvantaged communities. These areas often face a marked shortage of qualified staff or even their absence [27].

Notably, workforce planning via the WISN method is part of a strategic approach, insofar as the challenges observed in healthcare facilities do not arise solely from a shortage of staff. Similarly, increasing the number of healthcare professionals is not a universal solution to all difficulties. Analysis of the data used in our calculations also highlights other structural factors, such as the number of days of absence due to illness or various forms of leave, which contribute to increasing the pressure on midwives, particularly in the context of growing demand. Thus, workforce planning in the PHFs studied remains a strategic process aimed at achieving a balance between supply and demand on the basis of a rigorous assessment of the availability of staff and their work processes [13].

Tshis research offers valuable insights into the workload of healthcare providers in the era of free maternal healthcare in the DRC. To our knowledge, this is one of the first studies to document the issue of workload in this context and to identify the pressure exerted on midwives via the innovative WISN approach.

Our study has certain methodological limitations that could influence the reported WISN results. The main concern concerns the reliability of the annual statistics used, which are a central element of the WISN method. The study was based on individual attendance sheets, activity registers and service reports. In the DRC, these paper documents are often poorly preserved due to the weakness of the archiving management system. The study relied on individual attendance sheets, activity registers, WISN schedules, and service reports. In the DRC, these paper documents are often poorly preserved due to weaknesses in the archiving management system. Nevertheless, their rigorous exploitation, combined with systematic data triangulation, enabled us to obtain relatively reliable estimates of AWT and annual statistics on midwives workload. In addition, the verification of services by PUE-HSPF, as part of the strategic purchase of services, has limited the risk of overestimating these data.

## Conclusion

This study revealed significant work overload among midwives in the first- and second-level primary health care facilities studied in Kananga, due to an imbalance between workload and the number of staff available, as shown by the results of our WISN. This shortage of midwives could lead to occupational stress and affect their job satisfaction and motivation, which may compromise the quality of maternal care provided and limit the success of the free maternal healthcare policy. Consequently, this could hinder progress toward universal health coverage in the country. The Ministry of Public Health could institutionalize the WISN process for credible human resources for health planning. The WISN study could also be extended to secondary and tertiary levels of healthcare to better understand the workload of midwives. This would help guide decision-making and ensure the effectiveness of free maternal healthcare [40].

## Supplementary Information

Below is the link to the electronic supplementary material.

Supplementary Material 1

Supplementary Material 2

## Data Availability

The data that support the findings of this study are available from the corresponding author upon prior request.

## Abbreviations

ANC: Antenatal Consultation
AWT: Available Working Time
CAF: Category Allocation Factor
CKHC: Christ the King Health Center
DHS: Demographic and Health Survey
DRC: Democratic Republic of the Congo
PUE-HSPF: Public Utility Etablishment-Health Services Purchasing Fund
FP: Family Planning
GRH: General Referral Hospital
GSGRH: Good Samaritan General Referral Hospital
GSH: Good Shepherd Hospital
HC: Health Center
HF: Health Facility
UHC: Universal Health Coverage
HP: Health Post
HRH: Human Resources for Health
HZ: Health Zone
IAF: Individual Allocation Factor
KMSH: Kalemba Mulumba Secondary Hospital
LGRH: Lunkonga General Referral Hospital
MC: Management Committee
MU-HSDP: Management Unit of the Health System Development Program
MWHC: Mamu Wetu Health Center
NEGRH: Ndesha Etat General Referral Hospital
PHF: Primary Health Facility
PMTCT: Prevention of Mother-to-Child transmission
STHF: Second-Tier Healthcare facilities
