# Supplementary material for: Midwives workload in the context of free maternal healthcare: a cross-sectional study based on the Workload Indicators of Staffing Needs (WISN) method in primary healthcare facilities in Kananga, Democratic Republic of the Congo
Source: BMC Health Serv Res. 2025 Nov 14;25:1468. doi: 10.1186/s12913-025-13656-y (PMC12619488; doi:10.1186/s12913-025-13656-y)
Supplement: Supplementary file 1 — Supplementary Material 1 [file 12913_2025_13656_MOESM1_ESM.pdf]

DATA COLLECTION TOOLS

A. **Health Facility Identification**

- Facility Name : ………………………………………………………
- Level of Care : ……………………………………………………….
- Ownership Status : ☐ **Public** ☐ **Private** ☐ **Parastatal**

B. **Interview guide for midwives and human resources managers**

Supplemntary Table 1 : Available Work Time (AWT)

Headings	Workforce
On average, how many days per week did you work over the past year ?	
On average, how many hours per day did you work over the past year ?	
How many days did you spend in training over the past year ?	
How many days of annual leave did you take over the past year ?	
Number of public holidays	
Number of days of special leave/sick leave	
Number of days of unnotified special leave	
Number of training days per year	

C. **Activity Observation Guide**

Supplemntary Table 2 : Health Service Activities carried out by all midwives

Workload components	Number of minutes per woman ((determined with stopwatch))
Delivery (normal delivery)	Minutes/patient
Delivery (assisted)	Minutes/patient
Curative consultation	Minutes/patient
Antenatal clinic (ANC) — first visit	Minutes/patient
ANC — second visit	Minutes/patient
ANC — Third visit	Minutes/patient
ANC — Fourth visit	Minutes/patient
Post-natal care (booked case and unbooked) — First assessment	Minutes/patient
Post-natal care (booked case and unbooked) — Second assessment	Minutes/patient
Post-natal care (booked case and unbooked) — Third assessment	Minutes/patient
Family planning — counselled	Minutes/patient
Family planning — oral	Minutes/patient
Family planning — injectable	Minutes/patient
Family Planning — Introduction to the Use of Condoms	Minutes/patient
Family Planning — Introduction to the Use of the Menstrual Cycle Necklace Method	Minutes/patient
Family planning — insertion (IUCD and implant)	Minutes/patient
PMTCT — mothers (counselling for booked case and unbooked)	Minutes/patient

Supplemntary Table 3 : Support activities carried out by all midwives

Workload components	Effective working time	Unit time
Dressing changes		Day/week/month/year
Staff meetings		Day/week/month/year
Group health education		Day/week/month/year
Recording of Daily Data		Day/week/month/year
Handing over/taking over, report writing and ward round		Day
Service Verification by *Public Utility Etablishment (PUE)*		Day/week/month/year

Supplemntary Table 4 : Additional activities carried out by some midwives

Workload components	Number of staff performing the task	AWT	Unit time
General administrator			Day/week/month/year
Monthly Report Writing			Day/week/month/year
Mentoring of Subordinates			Day/week/month/year
Monitoring Meeting			Day/week/month/year
Meeting for the Review of Maternal Deaths			Day/week/month/year
Meeting of Department Heads			Day/week/month/year
Supervision of trainees			Day/week/month/year
