# Supplementary material for: Midwives workload in the context of free maternal healthcare: a cross-sectional study based on the Workload Indicators of Staffing Needs (WISN) method in primary healthcare facilities in Kananga, Democratic Republic of the Congo
Source: BMC Health Serv Res. 2025 Nov 14;25:1468. doi: 10.1186/s12913-025-13656-y (PMC12619488; doi:10.1186/s12913-025-13656-y)
Supplement: Supplementary file 2 — Supplementary Material 2 [file 12913_2025_13656_MOESM2_ESM.pdf]

Sumpplementary Table 5. How to round the factional results

Fraction	Rouding	Fraction	Rouding
1.0-1.1	Rounded down to 1	>1.1-1.9	Rounded up to 2
2.0-2.2	Rounded down to 2	>2.2-2.9	Rounded up to 3
3.0-3.3	Rounded down to 3	>3.3-3.9	Rounded up to 4
4.0-4.4	Rounded down to 4	>4.4-4.9	Rounded up to 5
5.0-5.5	Rounded down to 5	>5.5-5.9	Rounded up to 6
